# Clinical Significance of Polymorphisms in Immune Response Genes in Hepatitis C-Related Hepatocellular Carcinoma

**DOI:** 10.3389/fmicb.2019.00475

**Published:** 2019-03-15

**Authors:** Valli De Re, Maria Lina Tornesello, Mariangela De Zorzi, Laura Caggiari, Francesca Pezzuto, Patrizia Leone, Vito Racanelli, Gianfranco Lauletta, Laura Gragnani, Angela Buonadonna, Emanuela Vaccher, Anna Linda Zignego, Agostino Steffan, Franco M. Buonaguro

**Affiliations:** ^1^Centro di Riferimento Oncologico, Cancer Institute, Aviano, Italy; ^2^Istituto Nazionale Tumori IRCCS “Fondazione G. Pascale”, Naples, Italy; ^3^Department of Biomedical Sciences and Human Oncology, Section of Internal Medicine, University of Bari “Aldo Moro”, Bari, Italy; ^4^Department of Experimental and Clinical Medicine and Department of Oncology, Interdepartmental Hepatology Center MASVE, Azienda Ospedaliero-Universitaria Careggi (AOUC), Florence, Italy

**Keywords:** hepatitis virus C, hepatocellular carcinoma, cirrhosis, lymphoproliferative disorders, gene polymorphism, PD-1, IFNL3, TLR2

## Abstract

**Background and Aims:** Polymorphisms in the immune response genes can contribute to clearance of hepatitis C virus (HCV) infection but also mediate liver inflammation and cancer pathogenesis. This study aimed to investigate the association of polymorphisms in PD-1 (PDCD1), IFNL3 (IL28B), and TLR2 immune related genes in chronic HCV patients with different hepatic and lymphoproliferative HCV-related diseases.

**Methods:** Selected PDCD1, IFNL3, and TLR2 genes were tested by molecular approaches in 450 HCV-positive patients with increasing severity of underlying liver diseases [including chronic infection (CHC), cirrhosis and hepatocellular carcinoma (HCC)], in 238 HCV-positive patients with lymphoproliferative diseases [such as cryoglobulinemia and non-Hodgkin lymphoma (NHL)] and in 94 blood donors (BD).

**Results:** While the rs12979860 IFNL3 T allele was found a good marker associated with HCV-outcome together with the rs111200466 TLR2 del variant, the rs10204525 PD-1.6 A allele was found to have an insignificant role in patients with HCV-related hepatic disorders. Though in Asian patients the combination of IFNL3 and PD-1.6 markers better define the HCV-related outcomes, in our series of Caucasian patients the PD-1.6 A-allele variant was observed very rarely.

**Conclusion:** Differences in the incidence of HCV-related HCC and clinical response between Asians and Europeans may be partially due to the distribution of PD-1.6 genotype that we found divergent between these two populations. On the other hand, we confirmed in this study that the polymorphic variants within IFNL3 and TLR2 immune response genes are significantly associated with HCV-related disease progression in our cohort of Italian patients.

## Introduction

Hepatocellular carcinoma is the primary malignancy of the liver that often occurs in the setting of underlying chronic liver disease, mostly HBV and/or C virus infection (HBV and HCV, respectively), alcoholic liver disease, and non-alcoholic fatty liver disease. In the last years the incidence rate of HCC has increased in the European and American populations (Ryerson et al., 2016), mostly related to the increase of HCV infection acquired before the availability of the serologic test.

Curative treatment options for HCC are local resection, radioembolization and multikinase inhibitors. Available options in patients with unresectable HCC are liver transplantation, percutaneous ethanol injection, radiofrequency ablation, and transcatheter arterial chemoembolization (Hernaez and El-Serag, 2018).

Unfortunately, most patients have locally advanced or metastatic HCC at diagnosis and are not eligible for either liver resection or transplantation. In these cases, despite the attempt to improve the OS of patients by chemotherapy, radioembolization, and multikinase inhibitor sorafenib, the OS remains poor (Llovet et al., 2008). The role of tumor-infiltrating leukocytes in mediating cancer progression and efficacy of immunotherapy in other malignancies, like melanoma, are now well recognized. Thus, although liver represents an “immune privileged” organ, immunotherapy now quickly evolves as a treatment option for HCC (Prieto et al., 2015). Based on programmed cell death 1 (PD-1) and PD-L ligands checkpoint blockade, the immunotherapy for HCC has shown encouraging results in phase I/II trials of Nivolumab (Checkmate 040 trial) (El-Khoueiry et al., 2017).

The PD-1/PD-L pathway has been demonstrated to be engaged in the inhibition of activated T-cells with PD-1 up-regulated in exhausted CD8 T-cells, a mechanism involved in hepatic viral persistence. The PD-1 expression has been shown to associate with the development of HBV-related liver diseases and the prognosis of HCC patients (Zhang et al., 2010; Li et al., 2013; Li et al., 2016). A recent proteomic study analyzing HCC cancer-immune landscape across tumor, non-tumor, and peripheral blood cells demonstrated the existence of a cancer-immune gradient which become progressively suppressive from the non-tumor to the tumor microenvironment (Chew et al., 2017). Specifically, authors have demonstrated the importance of the immunosuppressive action caused by exhausted tumor-infiltrating memory CD8+ T cells expressing high levels of PD-1, that allows immune evasion by the virus and cancer cells (Blank et al., 2005; Park et al., 2015). The increase number of exhausted PD-1+ T-cells was significantly higher in HBV-related vs. non-viral-associated HCC, and much more increased during the HCC progression stage (stage 1 vs. stage ≥2) (Chew et al., 2017). PD-1 was also found significantly up-regulated in CD8+ cytotoxic T-cells in patients with chronic HCV-infection compared to either HCV-negative subjects or patients with spontaneous HCV resolution (Golden-Mason et al., 2007). *In vitro* blockade of PD-1 has been shown to restore the functional competence of the HCV-specific T-cells (Golden-Mason et al., 2007).

Two SNP on the chromosome 2 within the PDCD1 gene, the rs36084323 G/A (PD-1.1) located -606 base pairs upstream the promoter region at position 242801596 and the rs10204525 G/A (PD-1.6) located at +8669 base pairs in the 3′ UTR at the position 241850169, have been found to be significantly associated with the risk to develop HBV-related cirrhosis and HCC among a Chinese Han population (Zhang et al., 2010; Li et al., 2013; Peng et al., 2015). The mechanisms underlying this association are likely due to the rs36084323 G allele, positioned in a putative binding site for the UCE-2 transcription regulators, causing the increased expression of PD-1 (Sasaki et al., 2014), and the rs10204525 A allele, disrupting the binding sequence for miR-4717 inhibitor within the 3′ UTR of PD-1 mRNA, which drives increased PD-1 expression (Zhang et al., 2015). In fact, the miRNA-4717 was demonstrated to affect the luciferase activity in a dose-dependent manner in cells transfected with a recombinant vector expressing the luciferase reporter gene under the transcription control of the PD-1 promoter containing the rs10204525 G polymorphic variant (Zhang et al., 2015).

Hepatitis C virus leads to chronic hepatitis (CHC) and is a major cause of liver cirrhosis and HCC. HCV is also a lymphotropic virus that triggers B-cells and promotes favorable conditions for B lymphocyte proliferation, including the autoimmune condition MC and B-cell non-Hodgkin lymphoma (B-NHL) (De Re et al., 2007; Sansonno et al., 2007).

By exploring the relationship between innate immunity and HCV-related disorders we found that the IFNL3 C rs12979860 and TLR2 -196-174 ins polymorphisms, both associated with interferon-treatment response and spontaneous HCV-clearance as well as with lower HCV viral load, are associated with a decreased risk of HCV-related diseases and delay the occurrence of cirrhosis and HCC (De Re et al., 2016).

In the present study, we simultaneously analyzed the distribution of polymorphic variants in the PD-1, IFNL3, and TLR2 immune-related genes among Italian patients affected by HCV-related CHC, cirrhosis and HCC (*n* = 450) and we compared the genotype and allele frequencies with those obtained in patients affected by HCV-related lymphoproliferative diseases, such as MC and NHL, (*n* = 238) and in healthy BD (*n* = 94).

## Patients and Methods

### Study Design

A total of 148 HCV-infected patients with CHC without cirrhosis or HCC (48.3% male; median age 57.1 years), 113 patients with HCV-associated cirrhosis (65.4% males; median age 64.5 years), 189 patients with HCV-associated HCC (73.6% male; median age 68.9 years), 238 HCV-infected patients with lymphoproliferative disorders (130 MC, 29.1% male, median age 68.0 and 108 NHL, 47.5% male, median age 66.5 years), and 94 healthy BD (89.6% male; median age 42.5 years) were included in this study. Some of the individuals recruited for the study are part of a previous study [18]. Cases added as new are: BD *n* = 94, CHC *n* = 76, cirrhosis = 13, HCC = 102, MC = 130, NHL = 12. Demographic characteristics of the enrolled patients as well as HCV genotype and viral load were summarized in Table 1. Patients with CHC and healthy BD have a lower mean age. Female gender was more frequent among patients with MC.

**Table 1:** Clinical characteristics and PD-1.6 genotype of 688 HCV-positive patients and 94 HCV-negative BD.

						PD-1.6 A > G (rs10204525)				
	
	*n*	Age	Male (%)		MAF^∗^	G/G (%)	A/G (%)	A/A (%)	Viral load^#°^	HCV genotype° (%)
**Control subjects**										
BD	94	42.46 ± 10.1	43 (89.6)	94	0.10	76 (80.9)	18 (19.1)	0	–	–
**HCV infected patients with liver diseases**					
CHC	148	57.12 ± 14.1	56 (48.3)	450	0.09	125 (84.5)	20 (13.5)	3 (2.0)	2.32 ± 3.9	51/82 (55.6)
Cirrhosis	113	64.46 ± 11.1	70 (65.4)		0.09	93 (82.3)	19 (16.8)	1 (0.9)	2.71 ± 4.4	15/27 (62.2)
HCC	189	68.86 ± 8.6	103 (73.6)		0.09	154 (81.5)	34 (18.0)	1 (0.5)	1.94 ± 1.9	3/3 (100)
**HCV infected patients with lymphoproliferative diseases**					
MC	130	68.03 ± 9.9	32 (29.1)	238	0.11	103 (79.2)	26 (20.0)	1 (0.8)	3.32 ± 4.4	40/55 (72.7)
NHL	108	66.53 ± 15.1	47 (47.5)		0.10	87 (80.6)	20 (18.5)	1 (0.9)	3.13 ± 4.2	7/11 (63.6)
**Total**	782	65.9 ± 13.7	351 (44.9)	782	0.10	638 (81.6)	137 (17.5)	7 (0.9)	2.52 ± 3.9	116/178 (65.2)

*BD, blood donors; CHC, chronic hepatitis C; HCC, hepatocellular carcinoma; MC, mixed cryoglobulinemia; NHL, non-Hodgkin lymphoma; ±, standard deviation; ^∗^MAF, minor allele frequency, corresponding to A allele; ^#^viral load, mean in million; °data of viral load and of HCV genotype type 1 were determined in 178 cases*.

The diagnosis of chronic HCV infection was based on anti-HCV antibodies, elevated ALT serum levels and HCV RNA positivity for at least 6 months. The diagnosis of HCC was based on the standard criteria listed in the European Association for the Study of the Liver (EASL) that incorporate both invasive and non-invasive measures. Non-invasive criteria include two imaging techniques, both demonstrating a focal lesion >2 cm in diameter with features of arterial hypervascularization. Detection and immunochemical characterization of cryoglobulins were performed according to the consensus protocol proposed by the “Associazione Italiana per la Lotta alle Crioglobulinemie.” The diagnosis of NHL in the course of HCV infection has been histopathologically confirmed based on the WHO classification.

The study is in accordance with the principles of the Helsinki Declaration and all subjects provided written informed consent. The study was approved by institutional review boards and independent ethics committees since this was a multicenter study. Particularly, the study of HCC cases was approved by the ethical committee EUDRACT (No. 2010-023602-12), Comitato Etico Indipendente of the Azienda Ospedaliero-Universitaria “Consorziale Policlinico” di Bari, the scientific board and the ethics committee of the Istituto Nazionale Tumori “Fond Pascale”; the institutional review board code SPE 14.084_ AOUC; comitato Etico Area Vasta Centro AOU Careggi, Firenze.

The HCV antibody test was performed by an enzyme immunoassay (III-generation EIA) against HCV-core and HCV-non-structural antigens. The HCV viral load (RNA UI/mL) was assessed by branched DNA technology (Chiron, Emeryville, CA, United States) in serum samples of 201 patients at the time of diagnosis of the HCV-related disorder. HCV genotype was determined by a commercial, certified, diagnostic test (Versant HCV Genotype 2.0, Siemens Healthcare Diagnostics, Deerfield, IL, United States).

### Genotyping of PD-1, IFNL3, and TLR2 Polymorphisms

We collected 2 mL of whole blood from each patient and cryopreserved at -20°C until use. Total genomic DNA was extracted from peripheral blood using Qiagen DNAeasy Kit (QIAGEN, Grand Island, NY, United States). We analyzed four polymorphisms within the PD.1, IFNL3, and TLR2 genes, previously described as genetic factors involved in the immune response and hepatic disease progression (Zhang et al., 2010; Park et al., 2015; Li et al., 2016; Asian liver center, 2018; CDC, 2018). They include 3 single-nucleotide changes at positions -606 G/A (rs36084323, PD-1.1) (Xiao et al., 2015) and +8669 G/A (rs10204525, PD-1.6) within the PD-1 gene (Xiao et al., 2015), at position +1825 C/T (rs12979860) in the IFNL3 gene (De Re et al., 2016) and a 22-bp nucleotide del/ins from the position -196 to -174 (rs111200466) in the untranslated 5′-region of TLR2 gene (De Re et al., 2016).

Oligonucleotides used for genotyping were listed in Supplementary Table S1. Particularly, PD-1.1 and PD-1.6 were amplified as described by Zhang et al. (2010) by using PCR and products subjected to automated bidirectional direct sequencing analysis (Eurofins Genomics GmbH, Ebersberg, Germany). Briefly, PCR reactions were performed in 50 μL reaction mixture containing 30–300 ng of genomic DNA, 10 pmol of each primer, 1.25 Unit of Hot Master Taq DNA Polymerase (5 Prime GmbH, Hamburg, Germany) and 25 μL of PreMixJ (MasterAmp^TM^ PCR, Epicentre, Madison, WI, United States). DNA was amplified in Sure Cycler 8800 thermal cycler (Agilent Technologies, SantaClara, CA, United States) starting with an initial denaturation at 94°C for 3 min, followed by 30 amplification cycles of denaturation at 94°C for 30 s, annealing at 65°C for 30 s, elongation at 72°C for 1 and 10 min final elongation at 72°C. PCR amplification generated a fragment of 730 and 490 bp for the PD-1.1 and PD-1.6, respectively.

IFNL3 genotyping was performed using a specific custom TaqMan SNP-genotyping Assay (SNP rs12979860; Applied Biosystem, Foster City, CA, United States) on a 7900HT Fast Real-Time PCR system (Applied Biosystem, Foster City, CA, United States) (De Re et al., 2016). Determination of TLR2 polymorphism was performed by allele-specific PCR method. Fragments of different length (264 and 286 bp), depending on the presence or absence of the del mutation were visualized by electrophoresis on a 3.5% agarose gel stained with ethidium bromide (Supplementary Figure S1). Amplicon sequencing was used to validate the genotyping techniques.

### Statistical Analysis

Specific tests including Fisher’s exact test and one or two-way analysis of variance were used to compare allele and genotype frequency of PD-1, TLR2, and IFNL3 polymorphisms between patient groups with different pathologies and control subjects. Multivariate logistic regression analysis was performed with diagnosis as a dependent variable and independent variables, including age, gender (0 female; 1 male), and each genotype was also considered. *P*-value, OR and 95% CIs were calculated. Genotypes of each polymorphism were assessed according to dominant (0 wild-type homozygote; 1 heterozygote and variant homozygote), recessive (0 wild-type homozygote and heterozygote; 1 variant homozygote) and additive genetic models. Statistical power calculation was performed by using OSSE online tool^1^. Statistical analyses were performed using GraphPad Prism v6 and SNPStats. *P* value < 0.05 was considered statistically significant.

## Results

### Genotype Frequencies

The genotype and allele frequencies of PD-1.6 in HCV-related cases and healthy BD are listed in Table 1. Male gender was predominant in our cohort of BD (89.6%), due to psychological, cultural, and social reasons. The analysis of PD-1.6 genotype distributions among HCV-related cases, compared to that of BD showed no significant association with the risk of development of liver diseases or lymphoproliferative disorders.

The A-allele MAF PD-1.6 was 0.09 in patients with liver diseases, 0.10 in patients with lymphoproliferative disorders and 0.10 in BD. The frequency of PD-1.6 A/A genotype ranged between 0.5 and 2% in HCV-related cases; 0.8–0.9% in lymphoproliferative disorders and the allele A, and thus the genotype A/A, was not found among BD subjects. Differences in allele frequencies and genotype distribution between HCV-related diseases and BD were not statistically significant. By comparing the distribution of PD-1.6 alleles among all HCV-related liver diseases (CHC, cirrhosis, and HCC) with HCV-related lymphoproliferative disorders (MC, NHL) a significantly higher frequency of A allele was found in the latter group (83/817 and 50/426, respectively, *p* = 0.018). However, no statistically significant difference was observed by comparing the A allele distribution in HCV-related liver diseases or in HCV-related lymphoproliferative disorders with that determined in the BD group. Age and gender of BD did not affect the result of the study: chi-squared test for trend among individuals with <40; <50, and ≥50 years old was *p* = 0.74, 0.22, and 0.62 for PD-1, IFNL3, and TLR2, respectively; chi-square test for gender (female vs. male) was *p* = 0.67, 0.83, and 0.92 and for PD-1, IFNL3, and TLR2, respectively. The allele frequency and genotype distribution were also found independent of HCV viral load and HCV genotype (Table 1).

The PD-1.1 polymorphism was analyzed in 109 HCC cases and consistently with the allele frequency distribution in the Caucasian population all samples were found G/G homozygous for such polymorphism (data not shown).

The analysis of IFNL3 rs12979860 polymorphism was shown in Table 2. There was an increase of T allele frequency, showing an additive genotype trend, in patients with liver diseases, particularly CHC (OR = 1.57; 95% CI, 1.06–2.31), cirrhosis (OR = 2.10; 95% CI, 1.40–3.16), and HCC (OR = 1.79; 95% CI, 1.21–2.64) compared to BD controls. This analysis had 78% power to detect differences in IFNL3 C/T allele distribution.

**Table 2:** IFNL3 and TLR2 genotypes among 688 HCV-positive cases and 94 HCV-negative BD.

		IFNL3 C > T (rs12979860)					TLR-2 ins/del rs111200466)				
	*n*	C/C (%)	C/T (%)	T/T (%)	MAF^∗^	OR (95%CI)	Ins/Ins (%)	Ins/Del (%)	Del/Del (%)	MAF^∗^	OR (95%CI)^†^
**Control subjects**											
BD	94	42 (44.7)	47 (50.0)	5 (5.3)	0.30	Reference	72 (76.4)	21 (22.3)	1 (1.4)	0.12	Reference
**HCV infected patients with liver diseases**											
CHC	148	44 (29.7)	88 (59.4)	16 (10.8)	0.41	1.57 (1.1–2.3) *p* = 0.02	101 (68.2)	41 (27.0)	6 (4.7)	0.16	
Cirrhosis	113	26 (23.0)	66 (58.4)	21 (18.6)	0.48	2.10 (1.4–3.2) *p < 0.001*	82 (72.6)	26 (23.0)	5 (4.4)	0.16	
HCC	144	42 (29.2)	78 (54.2)	24 (16.7)	0.43	1.79 (1.2–2.6) *p* = 0.003	97 (67.4)	32 (22.2)	15 (10.4)	0.21	1.97 (1.2–3.3) *p* = 0.011
**HCV infected patients with lymphoproliferative diseases**											
MC	130	55 (42.3)	59 (45.4)	16 (12.3)	0.35		86 (66.2)	38 (29.2)	6 (4.6)	0.19	1.71 (1.0–2.9) *p* = 0.050
NHL	108	41 (38.0)	54 (50.0)	13 (12.0)	0.37		83 (76.9)	24 (22.2)	1 (0.9)	0.12	
**Total**	737	250 (33.9)	392 (53.2)	95 (12.9)	0.40		521 (70.7)	182 (24.7)	34 (4.6)	0.16	

*BD, blood donors; CHC, chronic hepatitis C; HCC, hepatocellular carcinoma; MC, mixed cryoglobulinemia; NHL, non-Hodgkin lymphoma; ^∗^MAF, minor allele frequency, corresponding to deletion (del) in TLR2 and T-allele in IFNL3; OR, odds ratio; 95% CI, 95% confidence interval; **^†^**only significant results have been reported*.

**Figure 1:**
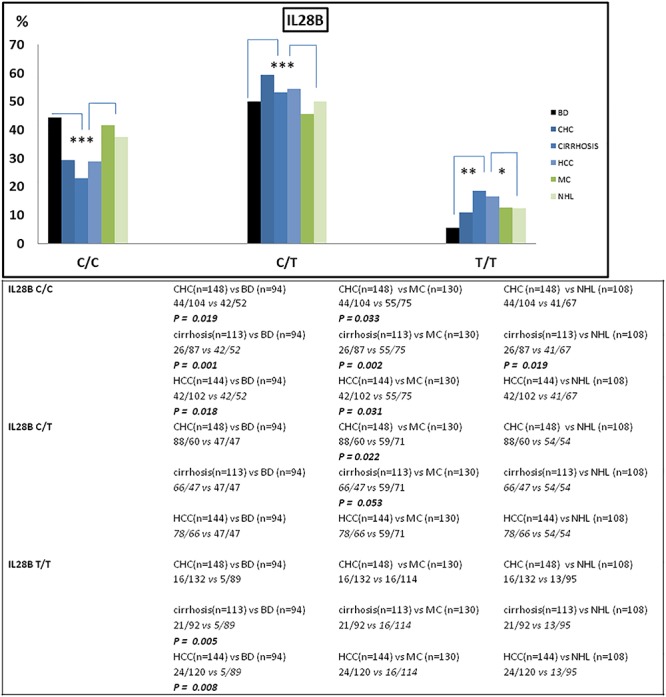
Frequency of IFNL3 genotype distribution among patients stratified on the basis of their HCV-related disease (*n* = 643) and blood donors (BD) (*n* = 94). The frequency of IFNL3 T allele was found increased in HCV-associated liver diseases (CHC, cirrhosis, and HCC) compared to BD and to lymphoproliferative disorders (MC and NHL). BD, blood donors (controls); CHC, chronic hepatitis C; HCC, hepatocellular carcinoma; MC, mixed cryoglobulinemia; NHL, non-Hodgkin lymphoma. ^∗^*p* < 0.05; ^∗∗^*p* < 0.001; and ^∗∗∗^*p* < 0.005.

The frequency of IFNL3 T allele was also higher in patients with hepatic diseases compared to the lymphoproliferative diseases (MAF 0.44 vs. 0.36; OR = 1.77, 95%CI 1.40–2.25, *p* < 0.0001). In particular, patients with more advanced HCV-related liver diseases (i.e., cirrhosis and HCC) the frequency of IFNL3 T/T homozygous genotype was 1.4-fold higher than in MC and NHL, and 3.3 higher than in BD (Figure 1 and Table 2). The IFNL3 T/T genotype was also 2.3-fold higher in MC and NHL patients compared to BD (Figure 1).

The distribution of TLR2 ins/del genotypes is shown in Table 2. The frequencies of these alleles in the HCV-related groups did not indicate any significant association, with the exception of the -196 to -174 del that was significantly more represented among HCC patients compared to BD controls (del vs. ins OR = 1.97; 95% CI, 1.17–3.31; *p* = 0.01). Moreover, we found a statistically significant difference in the frequency of del allele in HCV-related patients with MC lymphoproliferative disease compared to controls (OR = 1.71; 95% CI 1.00–2.91; *p* = 0.05).

Despite the limited power to detect the effect of TLR2 polymorphism, due to the low MAF, a statistically significant linear trend has been observed for TLR2 del/del genotype (Chi-square = 9.94, *p* = 0.0016) among the HCV-related groups.

### Epistatic Interaction Between IFNL3 and TLR2

The above results indicated that only the polymorphic variations in IFNL3 and TLR2 genes were associated with susceptibility to HCV-related diseases in our series (Tables 1, 2). A general linear regression model was used to identify multiloci genotypes associated with different HCV-related diseases. For the analysis, IFNL3-TLR2 genotypes from HCV-related liver and HCV lymphoproliferative diseases were divided into 4 groups, coded as InsC, InsT DelC, DelT and their frequencies were compared to those obtained in BD and lymphoproliferative vs. liver diseases (Table 3). Wild-type TLR2-ins- IFNL3-C was the most frequent group (Table 3). Some multilocus genotypes, i.e., ins-T and del-T distinguished patients with liver diseases compared to BD (OR = 1.68; 95% CI 1.06–2.65, *p* = 0.028) and lymphoproliferative vs. HCV-related liver disorders [(OR = 0.72; 95% CI 0.56–0.94), *p* = 0.014 and (OR = 0.43; 95% CI 0.22–0.85), *p* = 0.016, respectively], indicating that these multi loci genes play a significant role in the development of liver diseases among HCV-positive subjects.

**Table 3:** Comparison of TLR2 and IFNL3 multilocus genotypes frequencies of HCV-related patients with liver (*n* = 405), HCV-related lymphoproliferative diseases (*n* = 238) and blood donors (*n* = 94).

IFNL3	TLR2	BD	CHC	Cirrhosis	HCC	Hepatic diseases	Hepatic vs. BD OR (95%CI), p^†^	BD	MC	NHL	Lympho-proliferative	Lympho-proliferative vs. hepatic OR (95%CI), p^†^
C	Ins	0.58	0.47	0.45	0.45	0.45		0.58	0.54	0.53	0.53	
T	Ins	0.29	0.36	0.39	0.35	0.37	1.68 (1.1-2.7) *p = 0.028*	0.29	0.30	0.35	0.32	0.72 (0.6–0.9) *p = 0.014*
C	Del	0.10	0.11	0.10	0.13	0.11		0.10	0.12	0.10	0.11	
T	Del	0.03	0.07	0.07	0.07	0.07		0.03	0.05	0.02	0.04	0.43 (0.2–0.9) *p = 0.016*

*BD, blood donors (controls); CHC, chronic hepatitis C; HCC, hepatocellular carcinoma; MC, mixed cryoglobulinemia; NHL, non-Hodgkin lymphoma; ^†^only significant results were reported. OR, odds ratio; 95% CI, 95% confidence interval*.

Comparison of IFNL3 T-allele distribution between groups of patients affected by different HCV-related liver diseases and healthy BD underlined the role of such polymorphic variant as dominant key factor for the progression of cirrhosis to the most advanced liver diseases in our series (Table 3).

### PD-1.6 and IFNL3 MAF Frequencies in Different Countries

Surveys of HBV infection and the rate of HCC in different geographic regions showed a great disparity between Asian and other populations. In fact, the incidence of liver cancer is about nine-fold higher in Asians compared to white Americans suggesting that genetic polymorphisms and environmental risk factors may be responsible for such divergences (Asian liver center, 2018; CDC, 2018). Therefore, we compared the frequencies of PD-1.6 and IFNL3 polymorphisms in different countries reported in the NCBI database^2^ and the frequencies found in our series (Table 4). A significant difference in allele distribution among Asian and Italian population was observed both for the PD-1.6 (MAF 0.66 vs. 0.10) and IFNL3 (0.08 vs. 0.31) polymorphisms as shown in Table 4.

**Table 4:** Distribution of PD-1.6 allele-A and IFNL3 allele-T frequencies in different populations (available in http://www.ncbi.nlm.nih.gov/SNP/ database) and in our series of HCV-related diseases (*n* = 710).

	MAF	Asian	Nigerian	African	American	European	Italian	HCV^∗^
PD-1.6	A	0.66	0.50	0.39	0.39	0.12	0.10°	0.10
IFNL3	T	0.08	0.50	0.67	0.40	0.31	0.31^&^	0.40

^∗^*Data obtained from our series of patients with a chronic HCV infection (*n* = 710). °data obtained from our series of blood donors (*n* = 94). ^&^mean data from our series of HCV-negative patients (*n* = 94); *n* = 134 (Taliani et al., 2013); *n* = 428 (Falleti et al., 2011)*.

## Discussion

Previous studies have demonstrated that elevated expression of PD-1 in lymphocytes within the liver, especially exhausted T cells and Tregs, are closely associated with a dysfunction of the immune response in chronic HBV infection and HBV-related HCC (Boni et al., 2007; Fisicaro et al., 2010; Hsu et al., 2010; Wang et al., 2011). Moreover, it has been reported that PD1.1 and PD-1.6 polymorphisms combined with chronic HBV infection contribute to the development of HCC in a Chinese population (Li et al., 2013) and polymorphisms concur in the development of several tumor types and autoimmune disease pathogenesis (Momin et al., 2009; Liu et al., 2011; Tahoori et al., 2011; Li et al., 2013; Tang et al., 2017; Tejeda et al., 2017; Salmaninejad et al., 2018).

Studies focusing on CHC, by Penna et al. (2007) and Radziewicz et al. (2007) have shown that up-regulation of PD-1 affects HCV-specific CD8+ T cell function in the intrahepatic compartment in patient with chronic HCV infection. Blockade of the PD-1/PD-L1 interaction was shown to improve the expansion ability and IFN-γ secretion from HCV-specific CD8+ T cells (Moreno-Cubero and Larrubia, 2016) and control HCV replication in a chimpanzee model of CHC, although the efficacy was noted only in those animals with a critical threshold of pre-existing HCV-specific CD8+ T cells (Fuller et al., 2013). Additional studies showed that PD-1 is also critical in the persistence of chronic viral infections in mice (Barber et al., 2006) and in the progression of acquired immunodeficiency syndrome in humans (Day et al., 2006).

In our series we found a slight but not statistically significant increase of the PD-1.6 A/A genotype in the whole group of patients with HCV-related disorders compared to the control group of BD. However, the frequency of PD-1.6 A/A genotype is very limited, ranging from 0 to 2% (Table 1) resulting in a allele-A MAF of 0.10 (Table 4), while this unfavorable A/A genotype is the most common genotype (52.6%) in Asian population, with a allele-A MAF of about 0.66 (Table 4; Tang et al., 2017; Tejeda et al., 2017).

There are remarkable dissimilarities in the distribution of PD-1.6 polymorphic variants and their association with HCC between the Asian population and our Italian cohort. Since each of these studies comprised almost 1000 cases we are incline to think that differences in the PD-1.6 genotype distribution are consistent and reflect the genetic heterogeneity among various populations. On the contrary to Asian population, in our series we found a very low frequency of PD-1.6 A-allele variant (MAF 0.10, Table 4), thus it is hard to think that this mutation has a strong role in HCC in our population. Additionally, it is well known that persistent HBV infection were more likely to be associated with HCC in Asian population, while HCV infection had a higher prevalence among the Caucasian population (Ahmad et al., 2018; Falla et al., 2018). Further studies are needed to determine the distribution of PD-1.6 variants in different geographic regions and to explore their casual role in HCV-related diseases susceptibility worldwide.

Since the PD-1/PD-L1 blockade has proven to be an efficient treatment for HCC (Kudo, 2016), the lack of parallel changes in the frequency of PD-1.6 A allele in HCV-related HCC patients and controls in our series excludes a simple direct effect of PD-1.6 variant in the pathogenesis of HCC. However, we cannot exclude the possibility that other polymorphisms in PD-1 or in other immune-related genes, such as the rs12979860 polymorphism in IFNL3 gene, could be involved in HCV-related diseases in our Italian population (Ge et al., 2009; Riva et al., 2014; Wack et al., 2015). Alternatively, the discordant correlation between PD-1.6 and HCV-related HCC susceptibility across Asian-European populations could be related to an interaction of the host PD-1.6 gene variant with different environmental factor(s) present in the two populations or HCC development could be related to a different immune check point molecule blockade. Given the important involvement of PD-1 in autoimmunity and chronic viral infections, further researches are deserved to clarify the role of PD-1 polymorphism in these settings.

Genetic polymorphism of IFNL3 was found strongly associated with spontaneous resolution of HCV infection and with response to PEGylated interferon-alpha and ribavirin therapy for chronic HCV (Ge et al., 2009; Tanaka et al., 2009; Xiao et al., 2015; Huang et al., 2017). The IFNL3 and PD-1 markers in conjunction have also been reported to influence the susceptibility and outcomes of HCV infection in the Southeast China, suggesting their interactions in the disease outcomes (Xiao et al., 2015). In a previous study we found an association between TLR2 ins/del and IFNL3 polymorphisms with HCV-related outcome (De Re et al., 2016). In the present study we demonstrated that the multilocus TLR2-ins/ IFNL3 T genotype was a significant factor for development of HCV-related liver diseases (Table 3), and that the impact of rare PD-1.6 variant in Italian population is responsible for the discrepancy between Asian and European results (Table 4). In our series the IFNL3 T variant was confirming to be one of the best markers associated with HCV-related pathogenesis, with a marginal role of TLR2 del variant, while in Asian populations the combined IFNL3 and PD-1.6 polymorphisms were found to better define the HCV-related outcomes.

**Figure 2:**
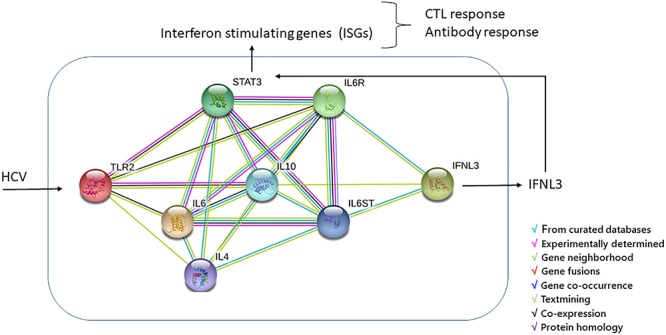
TLR2/IFN-λ3 protein-protein interaction was performed by using String software. The interleukin 6 (IL6) and INF-α, connecting TLR2 and IFN-λ3, can turn on the JAK/STAT pathway, the most important pathway in mediating the inflammatory response to HCV via induction of IFN-stimulated genes (ISGs). The final effect of ISGs results in antigen presentation and a differential expression profile of certain immunomodulatory genes targeting different immune cells and resulting in the polarization of Th1 and Th2 cells, in the modulation of regulatory T-cells and pro-inflammatory cytokines and in the differentiation of dendritic cells (DCs) from monocytes (Egli et al., 2014; Douam et al., 2017; Hemann et al., 2017; Syedbasha and Egli, 2017; Zhou et al., 2018).

Today, we have no data to demonstrate the effect of an interaction between TLR2 and IFNL3 gene products in HCV-positive patients, nonetheless, a functional links between these genes may be indirectly determined using the STRING^3^ software based on genomic associations of genes that are required for a same function. Figure 2 shows the graphical representation of the model of interaction between TLR2 and IFNL3 leading to effect of IFNL3 gene expression on the janus kinase (JAK)/signal transducer and activator of transcription (STAT) (JAK/STAT) pathway. HCV core and NS3 proteins are known to be able to trigger inflammatory pathways via TLR2, which may act, along with TLR1 and TLR6, as a receptor contributing to the activation of the innate immune system and production of interleukin 6 (IL-6) and Interferon-alpha (IFN-α) (Dolganiuc et al., 2004; Chang et al., 2007). In the past before direct-acting antiviral (DAA) treatment IFN-α therapy was largely demonstrated to reduce the risk of HCC and complications associated with cirrhosis in HCV infected individuals and serum IL-6 elevation has been correlated with liver disease severity, HCV-RNA titer and the activation of the JAK/STAT pathway (Malaguarnera et al., 1997; Sansone and Bromberg, 2012; Egli et al., 2014; Kong et al., 2016; Hemann et al., 2017; Syedbasha and Egli, 2017; Yakut et al., 2018). IFNL3 signal, producing IFN-λ3 molecules, has been demonstrated to inhibit HCV infection and induce anti-viral response also through the JAK-STAT pathway via induction of IFN-stimulated genes (ISGs) (Malaguarnera et al., 1997; Dolganiuc et al., 2004; Yakut et al., 2018). IFNL3 induces a cell type specific immune response due to the cellular expression of IFN-λ3s receptors in fewer cell types (Egli et al., 2014) and activates the JAK-STAT pathway by a feed-forward fashion with substantial differences in terms of the ISGs gene expression induced by IFN-α. Indeed, IFN-λ3 showed many antiviral properties but with an overall smaller response than IFN-α causes (Hemann et al., 2017; Syedbasha and Egli, 2017; Zhou et al., 2018). The IFN-λ3 effect is mainly associated with antigen presentation and a differential expression profile of certain immunomodulatory genes compared to IFN-α and this suggests a specific functional role for IFN-λ3. A critical role of IFN-λ3n in the polarization of Th1 and Th2 cells, in the modulation of regulatory T-cells and pro-inflammatory cytokines and in the differentiation of dendritic cells (DCs) have been well described in several reviews (Egli et al., 2014; Douam et al., 2017; Hemann et al., 2017; Syedbasha and Egli, 2017; Zhou et al., 2018). Of note, during infection with HCV, the expression pattern of many of the ISGs significantly change, most likely due to immunomodulatory effects of HCV proteins and complex inhibitory effects of IFN signaling pathways (Thomas et al., 2012; Egli et al., 2014). In particular, the long-term effects on the Th1/Th2 balance might have implications for the priming of T- and B-cell dependent memory responses, and thus possibly on HCV-related lymphoproliferative malignancy and autoimmune disease prevalence (Egli et al., 2014).

Thus, an indirect interaction between IFNL3 and TLR2 gene products may be suggested from data of literature, but further studies are necessary to confirm the effect of IFNL3 and TLR2 polymorphisms in the prediction of the above reported functional signaling in HCV patients.

This is the first study evaluating the PD1 polymorphisms and the risk of HCV-related disorders in the Italian population. The results should be regarded as descriptive observations and larger studies with more diverse ethnic populations are needed to confirm the association of immune related gene polymorphisms in HCV-related diseases.

In conclusion our study highlighted the importance of geographical difference in the frequencies of PD-1 and IFNL3 genetic polymorphisms in HCV-related diseases particularly in cirrhosis and in HCC susceptibility. Due to the importance of these genes in the immune response to hepatic infection, autoimmune disorders and malignancies as well as their role in the response to new proposed immune check-point treatment for HCC, further studies are needed to better understand the pathogenic role of these genetic variants in HCV-related diseases.

## Author Contributions

VD and MT wrote the manuscript, provided critical discussion in the manuscript preparation, and revised the manuscript. MD and FP performed the experiments and revised the manuscript. LC, PL, and LG contributed to analyze the data and revise the manuscript. VR, LG, AB, EV, AZ, AS, and FB contributed to collect and analyze the clinical patient’s data and revise the manuscript.

## Conflict of Interest Statement

The authors declare that the research was conducted in the absence of any commercial or financial relationships that could be construed as a potential conflict of interest.

## Footnotes

1 http://osse.bii.a-star.edu.sg/calculation2.php

2 http://www.ncbi.nlm.nih.gov/SNP/

3 https://string-db.org/cgi/input.pl?sessionId=7AG0SO9%20cCRu4&input%20_page_show_search=on

## Abbreviations

BD: blood donors
95% CI: 95% confidence interval
CHC: chronic infection
del: deletion
HBV: chronic hepatitis B
HCC: hepatocellular carcinoma
HCV: hepatitis C virus
ins: insertion
INFL: interferon lambda
MAF: minor allele frequency
MC: mixed cryoglobulinemia
NHL: non-Hodgkin lymphoma
OR: odds ratio
OS: overall survival
PCR: polymerase chain reaction
PD-1: programmed cell death protein 1
SNP: single nucleotide polymorphisms
TLR: toll like receptor
UTR: untranslated region.
