# Supplementary material for: Clinical Significance of Polymorphisms in Immune Response Genes in Hepatitis C-Related Hepatocellular Carcinoma
Source: Front Microbiol. 2019 Mar 15;10:475. doi: 10.3389/fmicb.2019.00475 (PMC6429030; doi:10.3389/fmicb.2019.00475)
Supplement: Supplementary file 1 [file Data_Sheet_1.pdf]

Suppl. table 1

Sequences of primers used for genotyping.

GENE	SNPs	Chromosome /position	Primers/Probes
PDCD1	rs36084323 (PD-1.1)	2/242801596 G>A	Forward primer: TCTAGCCTCGCTTCGGTTAT<br>Reverse primer: CTCACTGCTGTGGCCTCTTT<br>TCTAGCCTCGCTTCGGTTATTTTAAGCTGATGAGCCTGACGCATCTCATCACTAATATCAGCAGTTTCATTTCTCCTGTTTTC<br>CATTCGCTGTAATAAAATGCTCAGCACAGAATACAAGGAGATAAGCAAGCCATTTCACAAACGCCGGGCCGCCAGCCAGGCCC<br>AGGCACTGGACCCCCTGAACCACCCCACCCTGGCACGAGTGGGCTGGAGGGCAGGGCCCCGGGGAAGAAGGTCAAGGCTGGAA<br>GGGGAGGTCAGCCTCACAGCCAGCCCCTGCCACCGCCCCAGCCCCCCCGTCAGGCTGTTGCAGGCATCACACGGTGGAAAGAT<br>CTGGAACTGTGGCCATGGTGTGAGGCCATCCACAAGGTGGAAGCTTTGAGGGGGAGCCGATTAGCCATGGACAGTTGTCATTC<br>AGTAGGGTCACCTGTGCCCCAGCGAAGGGGGATGGGCC **[A/G]** GGAAGGCAGAGGCCAGGCACCTGCCCCCAGCAGGGGCAGA<br>GGCTGTGGGCAGCCGGGAGGCTCCCAGAGGCTCCGACAGAATGGGAGTGGGGTTGAGCCCACCCCTCACTGCAGCCCAGGAAC<br>CTGAGCCCAGAGGGGGCCACCCACCTTCCCCAGGCAGGGAGGCCCGGCCCCCAGGGAGATGGGGGGGATGGGGGAGGAGAAGG<br>GCCTGCCCCCACCCGGCAGCCTCAGGAGGGGCAGCTCGGGCGGGATATGGAAAGAGGCCACAGCAGTGAG
PDCD1	rs10204525 (PD-1.6)	2/241850169 G>A	Forward primer: GAAGTTTCAGGGAAGGTCAG<br>Reverse primer: CAGTGTGTGGATGTGAGGAG<br>GAAGTTTCAGGGAAGGTCAGAAGAGCTCCTGGCTGTGGTGGGCAGGGCAGGAAACCCCTCCACCTTTACACATGCCCAGGCAG<br>CACCTCAGGCCCTTTGTGGGGCAGGGAAGCTGAGGCAGTAAGCGGGCAGGCAGAGCTGGAGGCCTTTCAGGCCCAGCCAGCAC<br>TCTGGCCTCCTGCCGCCGCATTCCACCCCAGCCCCTCACACCACTCGGGAGAGGGACATCCTACGGTCCCAAGGTCAGGAGGG<br>CAGGGCTGGGGTTGACTCAGGCCCCTCCCAGCTGTGGCCACCTGGGTGTTGGGAGGGCAGAAGTGCAGGCACCTAGGGCCCCC<br>CAT **[A/G]** TGCCCACCCTGGGAGCTCTCCTTGGAACCCATTCCTGAAATTATTTAAAGGGGTTGGCCGGGCTCCCACCAGGGC<br>CTGGGTGGGAAGGTACAGGCGTTCCCCCGGGGCCTAGTACCCCCGCCGTGGCCTATCCACTCCTCACATCCACACACTG
IL28B	rs12979860	19/39248147 C>T	Custom TaqMan SNP-genotyping<br>Context Sequence [VIC/FAM]<br>TGAACCAGGGAGCTCCCCGAAGGCG **[C/T]** GAACCAGGGTTGAATTGCACTCCGC
TLR2	rs111200466	4/153684312 _153684334 del23	Forward primer: CTCGGAGGCAGCGAGAAA<br>Reverse primer: CTGGGCCGTGCAAAGAAG<br>CTCGGAGGCAGCGAGAAAGCGCAGC **[-/CAGGCGGCTGCTCGGCGTTCTCT]** CAGGTGACTGCTCGGAGTTCTCCCAGGTACG<br>TCGTGCGCTCCCCACTCGTGTGGTCTCTCTGCACCCCTTCCTGGGGTCGGGTCGGGCAGGGGCGGAGGGGAGCCGCAGCCCGG<br>TCACGGGCTCTGGGGAACCCGGGCTCCCGTTCGGCTGCACCTGGGCCCCTAGCTCCTGTCCGGGCGGGGATAGCGGGAAGCGC<br>ACCAGGCCCCCGGGACGCCGGTGCTTCTTTGCACGGCCCAG

SNPs single nucleotide polymorphisms

Suppl. Figure 1
Gel electrophoresis of rs111200466 TLR2 polymorphisms.

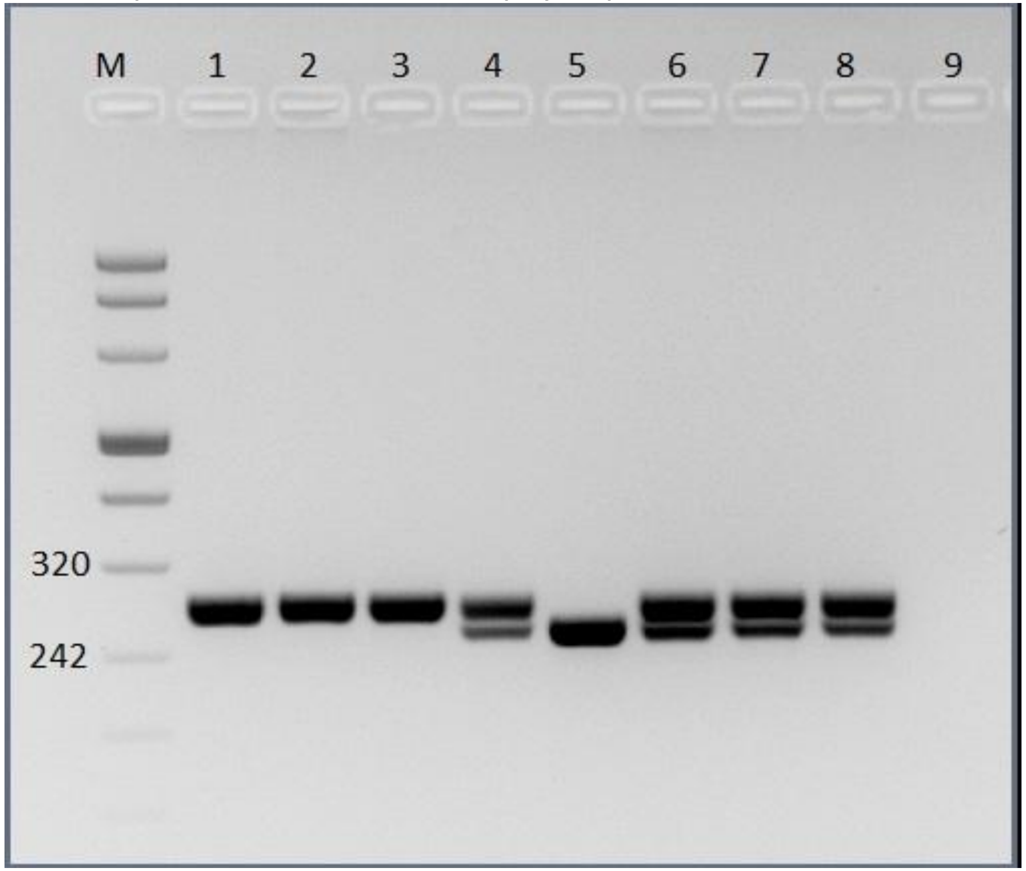

Agarose gel electrophoresis stained with ethidium bromide showing the amplification of the-196 to-174 del/ins polymorphism of toll-like receptor 2. M Marker, Lanes 1,2 and 3: PCR product of 286bp homozygous (ins/ins) genotype, Lanes 4, 6,7 and 8: PCR product of 286bp and 264bp heterozygous (ins/del) genotype, Lane 5: PCR product of 264bp homozygous (del/del) genotype, Lane 9 negative control.
